# Age and Sex Differences in Heart Rate Variability and Vagal Specific Patterns – Baependi Heart Study

**DOI:** 10.5334/gh.873

**Published:** 2020-10-21

**Authors:** Glaucylara Reis Geovanini, Enio Rodrigues Vasques, Rafael de Oliveira Alvim, José Geraldo Mill, Rodrigo Varejão Andreão, Bruna Kim Vasques, Alexandre Costa Pereira, Jose Eduardo Krieger

**Affiliations:** 1 Laboratory of Genetics and Molecular Cardiology, InCor-Heart Institute, Medical School, University of São Paulo, São Paulo, BR; 2 Laboratory of Medical Investigation LIM-37, University of São Paulo, Medical School, São Paulo, BR; 3 Postgraduate Program in Public Health, Federal University of Espírito Santo, Vitória, BR; 4 Department of Physiological Sciences, Health Science Center, Federal University of Espírito Santo, Maruípe, Vitória, BR; 5 Department of Electrical Engineering, Federal Institute of Espírito Santo, Vitória, BR; 6 Medical School-University Anhembi-Morumbi, São Paulo-Brazil, BR

**Keywords:** HRV, heart rate variability, cardiovascular diseases, aging, autonomic system, vagal tone

## Abstract

**Background:**

Heart rate variability (HRV) is a noninvasive method for assessing autonomic function. Age, sex, and chronic conditions influence HRV.

**Objectives:**

Our aim was to evaluate HRV measures exploring differences by age, sex, and race in a sample from a rural area.

**Methods:**

Analytical sample (n = 1,287) included participants from the 2010 to 2016 evaluation period of the Baependi Heart Study, a family-based cohort in Brazil. Participants underwent 24-hour Holter-ECG (Holter) monitoring. To derive population reference values, we restricted our analysis to a ‘healthy’ subset (i.e. absence of medical comorbidities). A confirmatory analysis was conducted with a subgroup sample that also had HRV derived from a resting ECG 10’-protocol obtained during the same time period.

**Results:**

The ‘healthy’ subset included 543 participants. Mean age was 40 ± 14y, 41% were male, 74% self-referred as white and mean body-mass-index was 24 ± 3kg/m^2^. Time domain HRV measures showed significant differences by age-decade and by sex. Higher values were observed for males across almost all age-groups. Parasympathetic associated variables (rMSSD and pNN50) showed a U-shaped distribution and reversal increase above 60y. Sympathetic-parasympathetic balance variables (SDNN, SDANN) decreased linearly by age. Race differences were no significant. We compared time domain variables with complete data (Holter and resting ECG) between ‘healthy’ versus ‘unhealthy’ groups. Higher HRV values were shown for the ‘healthy’ subset compared with the ‘unhealthy’ group.

**Conclusion:**

HRV measures vary across age and sex. A U-shaped pattern and a reversal increase in parasympathetic variables may reflect an age-related autonomic dysfunction even in healthy individuals that could be used as a predictor of disease development.

## Introduction

Heart rate variability (HRV) is a noninvasive method used to assess the autonomic nervous system (ANS) [1]. Briefly, it is defined as variations in both instantaneous heart rate and normal-to-normal (NN) intervals on an electrocardiogram (ECG); an abnormal HRV reflects dysregulation between the sympathetic and parasympathetic arms of the ANS [1]. In addition, reduced HRV is associated with adverse cardiovascular outcomes and mortality [2,3,4,5,6]. Previous studies have highlighted the prognostic information on risk of cardiac events provided by HRV measurements beyond that derived from traditional cardiovascular risk factors [2,3,4,5,6]. For instance, a decrement in the standard deviation of total normal NN intervals has been associated with a hazard ratio of 1.47 for new cardiac events [2]. Furthermore, HRV is sex- and age-related, being modulated by the aging process [7,8,9]. Although the aging process is associated with a decrease in HRV measures, some findings suggest that a decrease in HRV depends on the preservation of autonomic function, especially in the HRV-parasympathetic arm, instead of an age-related decrease [10]. How aging modulates autonomic function is still unclear. Indeed, the dysfunction of the ANS may display differences in the sympathetic and parasympathetic arms by age and HRV behavior according to aging and disease still needs clarification. Because age, sex, and disease influence HRV measurements, population reference values are mandatory, but there are discrepancies when studying different populations [1,9].

Therefore, to understand HRV measurements in a rural sample, the aim of this study is to evaluate HRV measures and differences by age, sex, and race, and describe population reference values using a long-term 24-hour ECG (Holter).

## Methods

### Study population

Participants from the second evaluation-wave (period from 2010 to 2016) of the Baependi Heart Study, a family-based cohort conducted in rural Brazil, who underwent Holter monitoring were our main sample (n =1,287). Baependi is a town in a rural area (752 km^2^, 18,307 inhabitants in the 2010 census) located in the state of Minas Gerais, Brazil [11]. The Baependi Heart Study was set up in 2005 to develop a longitudinal family-based cohort study to evaluate genetic and environmental influences on cardiovascular risk factor traits. Baependi is a town with very limited migration and a cohesive culture. The cohort characteristics were published before [11]. Due to the fact that several chronic conditions impact HRV measurements [2,3,4,5,6], we restricted our analysis to the subset characterized as ‘healthy.’ For this, we excluded those who self-reported any of the following conditions: hypertension, diabetes, stroke, cancer, myocardial infarction, cardiac revascularization surgery, angioplasty, those receiving a medication for hypertension, diabetes, dyslipidemia, those taking a beta-blocker, current smokers, and obese subjects (body mass index-BMI ≥30 kg/m^2^). After exclusions, the analytical sample comprised 543 participants. Demographics, anthropometrics, and 12h-fasting blood for lipid and glucose profiles were collected and analyzed by trained technicians. Twenty-four-hour ambulatory blood pressure measurement was recorded in the same time period.

The study protocol was approved by the ethics committee of Hospital das Clinicas, University of São Paulo-Brazil (approval number 0494/10), and each subject provided written informed consent before participation. The study protocol conforms to the ethical guidelines of the 1975 Declaration of Helsinki as reflected in a priori approval by the institution’s human research committee.

### HRV from Holter

Long-term 24-hour ECGs were recorded by using a 3-channel Holter system (Cardiolight, CardioSmart Office CS-530-CARDIOS) over a period of 24 hours during daily activities. Recommendations in the Guidelines of the Task Force of the European Society of Cardiology and the North American Society of Pacing and Electrophysiology measurements of the NN intervals for HRV analysis were followed [1]. Time domain variables were then calculated. We did not have access to frequency domain data, which is a study limitation. The recordings were analyzed by an experienced cardiologist. Non-sinus rhythms were excluded (nine participants because of atrial fibrillation/atrial flutter and three pacemaker users). Time domain analyses calculate the intervals between successive normal QRS complexes. The most common HRV measures are the standard deviation of all NN intervals (SDNN) and the squares of differences between adjacent NN intervals (rMSSD) [12]. HRV measures in this study were SDNN; standard deviation of the average of all consecutive 5-minute NN intervals (SDANN); mean of the standard deviations of all normal sinus NN intervals for all 5-min segments (SDNN index); rMSSD; percentage of consecutive NN intervals that deviate from one another by more than 50 ms (pNN50); and the triangular interpolation of NN intervals (TINN) [1,13]. For analysis purposes, we chose the measures that exhibit autonomic modulation: rMSSD and pNN50 as proxy for parasympathetic modulation and SDNN and SDANN mostly associated with sympathetic modulation or autonomic balance [1,14]. There is still concern about the effects of breathing on HRV measures. For the aim of this study, we chose not to address these technical limitations, but because it is known that the rMSSD is not affected by respiration patterns [15], we may use these measurements as a control variable ‘protected’ against this technical limitation.

### HRV from resting ECG10’-protocol

The sympathetic and parasympathetic activities were noninvasively estimated using time and frequency domain HRV measures. Heart rate was recorded by using a resting ECG signal with the subject in supine and orthostatic position for about 10 minutes in each position. The ECG recording was obtained with a digital device (Micromed-Brazil) at a sampling rate of 1 KHz. Software (WinCardio, version 4.4a, Micromed-Brazil) generated the series of R-R intervals (D-II lead) to be employed for HRV analysis. Detailed information on the R-R series filtering and processing were published elsewhere [16,17]. Briefly, HRV analysis was carried out in the time and frequency domains using Matlab-customized software. The R-R series were automatically processed to remove artifacts and ectopic beats, which were replaced by linear interpolation. Criteria used to select the 5-min interval for HRV analysis were previously published [16]. Time domain components included the average of all NN intervals, the variance of all NN intervals, SDNN, pNN50, and rMSSD. For power spectral analysis, the R-R series were processed by the autoregressive method (model of order 16) to identify the three main components of the periodic fluctuations of heart beats: the very-low frequency (VLF: 0-0.04 Hz), low frequency (LF: 0.04-0.15 Hz), and the high frequency (HF: 0.15-0.40 Hz) bands of the overall spectrum. Given that short term ECG recordings are inappropriate for investigating the VLF band, we only submitted LF and HF components in normalized units (nu) to analytic procedures. Normalization consisted of dividing the power of each component by total power minus power of VLF component. LF/HF ratio was used to indicate sympathovagal balance.

### Data analysis

Continuous variables are shown as mean and SD and categorical as percentages. HRV measurements are shown as age-decade (10-year age group) [9]. Age-decade groups were (18 to < 30y; 30 to < 40y; 40 to < 50y; 50 to < 60y; and ≥ 60y). We performed a 2-way ANOVA while analyzing sex differences according to the age-decade distribution. The same was done for race. We further used Locally Weighted Scatterplot Smoothing (LOESS) regression to account for nonlinearity in our data. We did a secondary and confirmatory analysis using data from a resting ECG10’-protocol (lying and standing) performed during the same time period. First, we explored HRV measures in the full sample of the resting ECG. Then, we selected only those classified as ‘healthy,’ using the same exclusion criteria as before. We analyzed the LOESS regression curves in this ‘healthy’ subset as well. We analyzed both time and frequency domain measures while using the resting ECG, but only time domain HRV measures using Holter data. Therefore, for comparison purposes between 10’ and 24-hour protocols, we addressed only time domain variables. Finally, we restricted group comparisons using only participants with complete data for all variables used. We compared groups (healthy vs. unhealthy) by age-decade testing for group differences. Data were analyzed using IBM SPSS statistical software version 18 and R package software version 3.5.2. The alpha level of significance was set as < 0.05.

## Results

**Figure 1:**
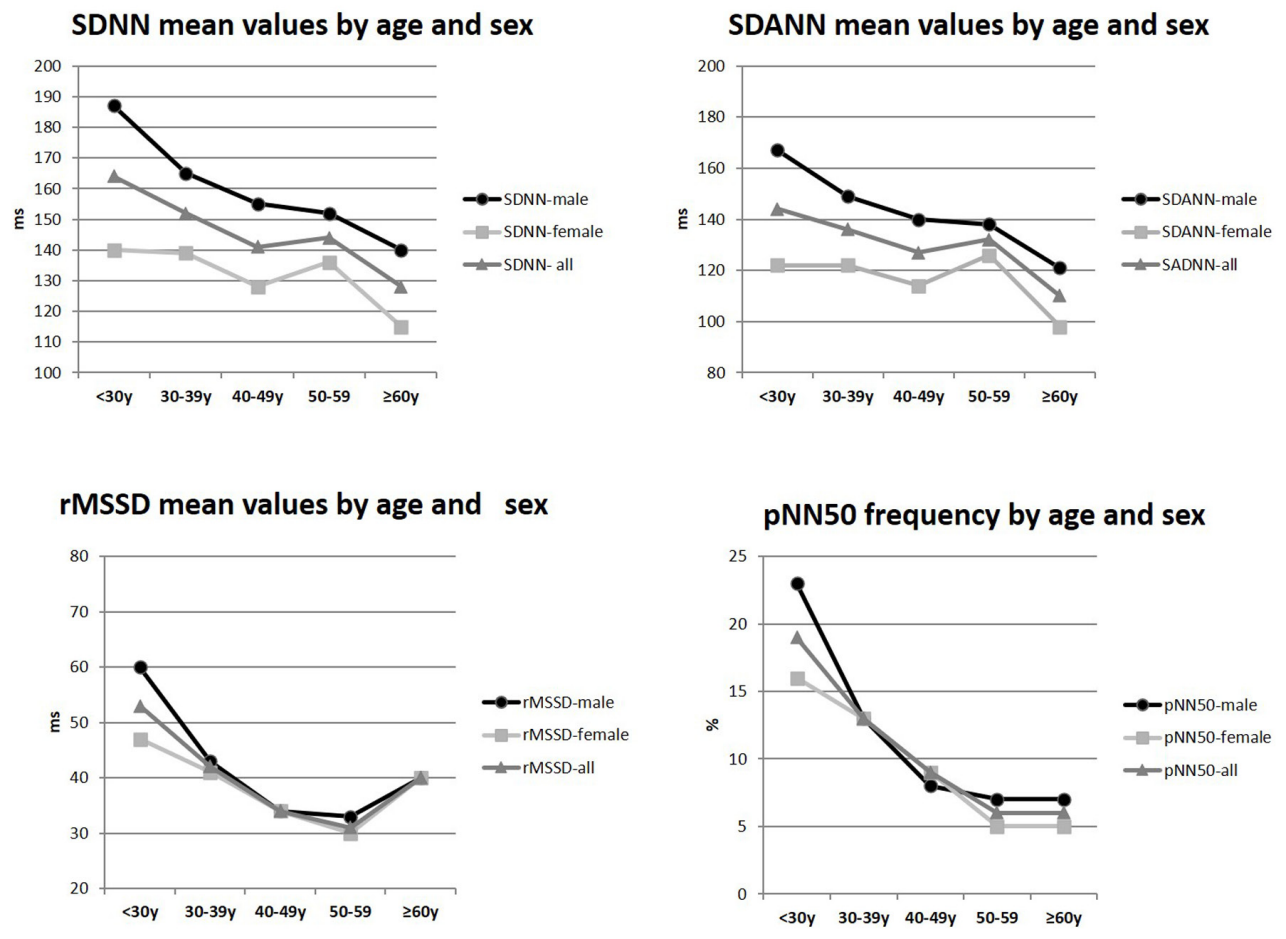
**HRV measures showing mean values by age decade and sex distribution of the ‘Healthy’ sample.** SDNN = standard deviation of NN intervals; SDANN = standard deviation of the average of all consecutive 5-minute NN intervals; rMSSD = root mean square of successive difference of NN intervals; pNN50 = percentage of consecutive NN intervals that deviate from one another by more than 50 ms. Female (n = 318) and Male (n = 225). Age ≥ 18 and < 30y (n = 137); age 30–39y (n = 154); age 40–49y (n = 110); age 50–59y (n = 92); age ≥ 60y (n = 50). According to 2-way ANOVA, testing difference among groups, for SDNN (p-value for sex = < 0.001, for age-decade = < 0.001 and for age-decade*sex interaction = 0.023); For SDANN (p-value for sex = < 0.001, for age-decade = < 0.001 and for age-decade*sex interaction = 0.011; For rMSSD (p-value for sex = 0.070, for age-decade < 0.001 and for age-decade*sex interaction = 0.096); For pNN50 (p-value for sex = 0.017, for age-decade = < 0.001 and for age-decade*sex interaction = 0.009).

**Figure 2:**
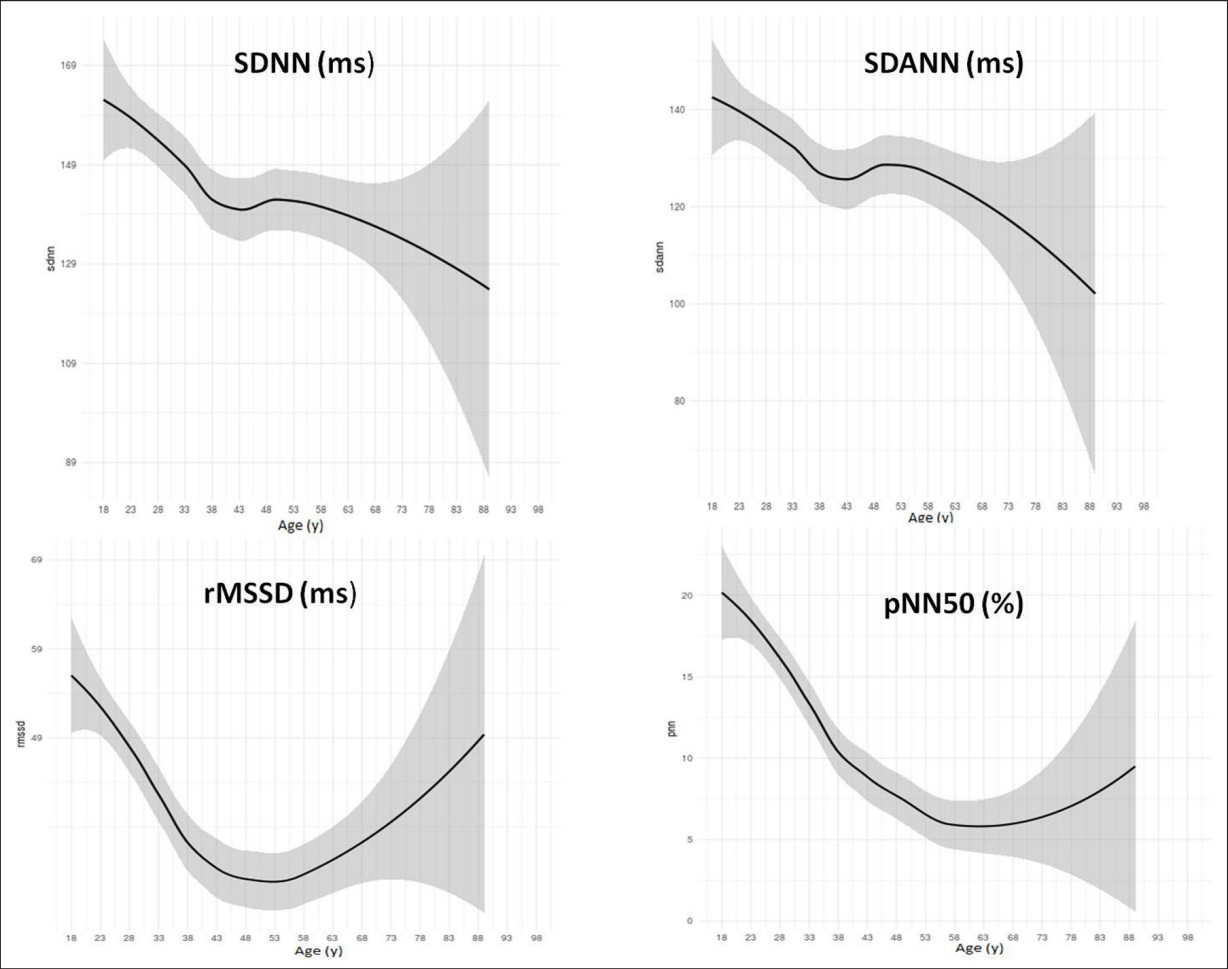
**HRV measures showing the LOESS regression curves by 5-year interval of the ‘Healthy’ sample (n = 543).** HRV = heart rate variability; LOESS (Locally Weighted Scatterplot Smoothing) regression curve, where the center is the predictive value and shadow represents 95% confidence intervals; SDNN = standard deviation of NN intervals; SDANN = standard deviation of the average of all consecutive 5-minute NN intervals; rMSSD = root mean square of successive difference of NN intervals; pNN50 = percentage of consecutive NN intervals that deviate from one another by more than 50 ms.

Figure A1 (Appendix) shows the exclusion criteria used to define our ‘healthy’ sample. The ‘healthy’ sample (n = 543) had a mean age of 40 ±14y, 41% male, 74% white, mean BMI of 24 ±3 kg/m^2^, and 83% never-smokers (Table 1). Table 1 also shows univariate comparison between sex and race groups (female vs. male; white vs. non-white). In general, males were older, smoked more, had higher levels of blood pressure and creatinine, but lower HDL levels than females. White participants were older, with lower HDL and higher HbA1c levels than non-white subjects, according to race groups. Of note, the general characteristics of the entire cohort (n =1,287 subjects), from which the ‘healthy’ subset was derived, are summarized in the Appendix, Table A1. The full sample was also predominantly female, but older than the ‘healthy’ subset. There was no difference according to mean 24-hour blood pressure between the ‘healthy’ and the full sample. Supplemental table A2 displays measurements from the Holter exam according to mean values of HRV variables for the full sample used to derive the ‘healthy’ subset. In general, HRV from the ‘healthy’ subset had higher mean values than those from the full sample (SDNN = 146 ± 37 vs. 138 ± 41ms; SDANN = 130 ± 39 vs. 123 ± 39ms; rMSSD = 41 ± 21 vs. 40 ± 27ms; pNN50 = 12 ± 10 vs. 10 ± 10%; respectively from ‘healthy’ subset vs. full sample). Table 2 shows HRV values by percentiles, means and standard error, and mean difference by sex (male – female). The findings in Table 2 show that SDNN, SDANN, and pNN50 reached statistical significance for sex-related differences (*P* < 0.001, *P* < 0.001, and *P* = 0.017, respectively), showing higher values for males than females (mean values for age group 40–49y, respectively male vs. female: SDNN = 155 vs. 128ms; SADNN = 140 vs. 114ms; and for age group 18–30y, pNN50 = 23 vs. 16%). rMSSD did not reach statistical significance (*P*-value = 0.07) for sex-related differences, but also showed nominally higher values for males than females (mean rMSSD for age group 18–30y, respectively male vs. female = 66 vs. 47 ms). We show sex-related differences by age-decade groups in Figure 1. SDNN and SDANN have a linear decrease with increasing age, except for the 50–59y age-group. rMSSD and pNN50 show a reversal increase above 60y (Figure 1). We then analyzed the LOESS regression curves by 5-year age interval for each HRV measure (Figure 2). The mean values for SDNN and SDANN in Figure 1 also showed the same pattern of linear decrease with increasing age. Interestingly, there is a U-shaped pattern for rMSSD and a reversal increase for pNN50. The nadir of the U-shape is around 53y and the reversal increase is over 60y. While testing differences by race (white vs. non-white), the white group had higher HRV measures than the non-white group for both males and females (SDNN in age-decade group 40–40y, respectively for white vs. non-white, for males = 157 vs. 150 ms, while for females = 127 vs. 127ms; for those over 60y, for males = 142 vs. 122ms and for females = 118 vs. 101ms). However, race-related differences were not statistically significant (Appendix, Figures from A2 to A4).

**Table 1:** Baseline characteristics of the healthy sample according to sex and race.

	All ‘Healthy’ sample (n = 543)	Female (n = 318)	Male(n = 225)	*P*-value	white(n = 402)	non-white (n = 141)	*P*-value

**Age, y**	40 ± 14	38 ± 13	43 ± 15	< 0.001	41 ± 15	37 ± 12	**0.004**
**Male, %**	41	–	–	–	42	40	0.615
**Race, %**							
White	74	73	75	0.908	–	–	–
Black	6	6	5				
Other	20	21	20				
**Smoking, %**							
Never	83	89	75	**< 0.001**	81	89	**0.040**
Former	17	11	25		19	11	
Current	0	0	0		0	0	
**Mean 24h-Systolic BP, mmHg**	116 ± 9	114 ± 9	120 ± 9	**< 0.001**	117 ± 9	116 ± 9	0.585
**Mean 24h-Diastolic BP, mmHg**	73 ± 7	71 ± 7	75 ± 8	**< 0.001**	73 ± 8	72 ± 6	0.625
**BMI, kg/m^2^**	24 ± 3	24 ± 3	24 ± 3	0.190	24 ± 3	24 ± 3	0.790
**Waist circumf, cm**	86 ± 10	86 ± 11	87 ± 8	0.190	86 ± 10	86 ± 9	0.484
**Hip circumf, cm**	96 ± 10	97 ± 12	95 ± 6	**0.027**	96 ± 11	98 ± 7	**0.034**
**Neck circumf, cm**	34 ± 3	33 ± 2	37 ± 3	**< 0.001**	35 ± 3	34 ± 3	0.447
**Total cholesterol, mg/dL**	195 ± 43	195 ± 41	196 ± 44	0.924	195 ± 41	196 ± 45	0.850
**LDL, mg/dL**	123 ± 37	120 ± 36	126 ± 38	0.103	123 ± 86	121 ± 40	0.645
**HDL, mg/dL**	49 ± 12	52 ± 12	44 ± 9	**< 0.001**	48 ± 12	51 ± 12	**0.008**
**Triglicerydes, mg/dL**	119 ± 60	116 ± 54	124 ± 68	0.157	121 ± 61	116 ± 58	0.400
**Glicose, mg/dL**	87 ± 10	86 ± 10	87 ± 10	**0.009**	87 ± 10	87 ± 9	0.917
**HbA1c,%**	5.35 ± 0.50	5.30 ± 0.50	5.40 ± 0.50	0.060	5.40 ± 0.50	5.20 ± 0.50	0.004
**Creatinine, mg/dL**	0.84 ± 0.17	0.80 ± 0.15	0.90 ± 0.15	**< 0.001**	0.83 ± 0.16	0.84 ± 0.17	0.344

Data are shown as mean ± SD for continuous and percentages for categorical variables. *P*-value by independent t-test for continuous and by chi-square for categorical variables. BP = blood pressure; BMI = body mass index, chol = cholesterol; LDL = low-density lipoprotein; HDL = high-density lipoprotein; HbA1c = glycated hemoglobin. See Appendix Figure A1 for detailed information about characterization of the ‘Healthy’ sample.

**Table 2:** HRV Measurements of the Restricted Analytical Sample (those designated as ‘Healthy’), According to Age- and Sex-Related Distribution by Percentiles.

HRV(n = 543)	n	Sex	Age	Percentile						Mean sex-difference(Male – Female)	*P*-value for sex-related difference

			Years	5^th^	25^th^	50^th^	75^th^	95^th^	Mean ± se		

**SDNN, ms**	50	**Male**	**18–30**	116	151	177	226	280	187 ± 6	28 ± 4	**< 0.001**
	54		**30–39**	114	134	159	190	240	165 ± 6		
	43		**40–49**	98	128	140	188	218	155 ± 7		
	43		**50–59**	85	122	149	180	218	152 ± 7		
	35		**≥ 60**	60	116	136	152	276	140 ± 7		
	87	**Female**	**18–30**	94	123	143	157	179	140 ± 3		
	100		**30–39**	89	120	137	158	197	139 ± 3		
	67		**40–49**	74	112	126	145	184	128 ± 4		
	49		**50–59**	77	121	136	155	186	136 ± 4		
	15		**≥ 60**	79	90	110	128	179	115 ± 8		
**SDANN, ms**	50	**Male**	**18–30**	97	130	153	209	256	167 ± 5	26 ± 3	**< 0.001**
	54		**30–39**	87	121	144	172	221	149 ± 5		
	43		**40–49**	81	112	126	171	215	140 ± 5		
	43		**50–59**	71	106	138	163	205	138 ± 5		
	35		**≥ 60**	51	100	120	132	234	121 ± 6		
	87	**Female**	**18–30**	77	104	125	138	160	122 ± 4		
	100		**30–39**	73	98	120	144	186	122 ± 4		
	67		**40–49**	57	93	111	138	162	114 ± 4		
	49		**50–59**	67	108	127	144	178	126 ± 5		
	15		**≥ 60**	60	77	93	113	152	98 ± 9		
**rMSSD, ms**	50	**Male**	**18–30**	27	44	53	81	106	60 ± 3	4 ± 2	0.070
	54		**30–39**	22	30	40	51	83	43 ± 3		
	43		**40–49**	17	24	32	45	56	34 ± 3		
	43		**50–59**	19	23	30	39	58	33 ± 3		
	35		**≥ 60**	12	22	29	51	153	40 ± 3		
	87	**Female**	**18–30**	24	34	45	58	78	47 ± 2		
	100		**30–39**	18	29	37	49	80	41 ± 2		
	67		**40–49**	20	25	31	39	64	34 ± 2		
	49		**50–59**	17	21	27	35	67	30 ± 3		
	15		**≥ 60**	16	25	29	36	105	40 ± 5		
**pNN50, %**	50	**Male**	**18–30**	5	13	22	33	46	23 ± 1	2 ± 1	**0.017**
	54		**30–39**	2	6	9	19	32	13 ± 1		
	43		**40–49**	1	3	6	12	20	8 ± 1		
	43		**50–59**	1	3	5	9	17	7 ± 1		
	35		**≥ 60**	0.25	1	3	7	38	7 ± 2		
	87	**Female**	**18–30**	4	10	15	21	32	16 ± 1		
	100		**30–39**	1	5	11	18	35	13 ± 1		
	67		**40–49**	1	3	7	12	31	9 ± 1		
	49		**50–59**	0.50	2	3	7	14	5 ± 1		
	15		**≥ 60**	1	1	4	6	13	5 ± 2		

‘Healthy’ sample excluded those under medication use, those who had HTN, DM, obesity, current smokers, past stroke, past AMI, cancer, cardiac revascularization or angioplasty (see Appendix Figure A1). Male (n = 225) and Female (n = 318). se = standard error; HTN = hypertension; DM = diabetes mellitus; AMI = acute myocardium infarction; NN = normal-to-normal; SDNN = standard deviation of NN intervals; SDANN = standard deviation of the average of all consecutive 5-minute NN intervals; rMSSD = root mean square of successive difference of NN intervals; pNN50 = percentage of consecutive NN intervals that deviate from one another by more than 50 ms.

We then conducted an analysis using data from the resting ECG10’-protocol. We first displayed the behavior of each HRV measure according to mean value in lie-down and standing positions for time and frequency domain variables (Appendix, Figures A5 and A6). Then, for further comparisons, we displayed HRV measures by age-decade groups for the time domain (Appendix, Figure A7) and the frequency domain (Appendix, Figure A8). In general, all HRV decreased as age increased, except for the HF measure, which increased as age increased. Although in the lie-down position, time domain variables decreased with increasing age, shown as a linear pattern, in the standing position, rMSSD and pNN50 showed a reversal increase over 60y. Frequency domain measures did not change whether in lie-down or standing positions, showing a pattern of decrease with increasing age (LFnu and LF/HF ratio) and the inverse association, increase with increasing age (HFnu). We restricted analysis to those classified as ‘healthy’ using the same criteria as in Appendix Figure A1. We got 538 subjects in this ‘healthy’ subset sample. We then compared the LOESS regression for both ‘healthy’ samples: those who underwent resting ECG vs. those who underwent Holter. Appendix Figures A9, A10 and A11 display LOESS curves for both ‘healthy’ samples. The SDNN showed a similar linear decrease pattern as age increased in both resting ECG and Holter (Appendix, Figure A9). rMSSD had shown the U-shaped pattern in Holter, but the same pattern was not shown for the resting ECG sample (Appendix, Figure A10). The reversal increase pattern in pNN50 demonstrated in the Holter group did not occur for the resting ECG sample (Appendix, Figure A11); however, while in mean values and the standing position (Appendix, Figure A7), both rMSSD and pNN50 showed the reversal increase pattern over 60y. Finally, we selected all complete data of the Holter and resting ECG and split into ‘healthy’ and ‘unhealthy’ groups (Appendix, Table A3), using similar criteria as listed in Appendix Figure A1. Holter HRV values were higher than resting ECG values (SDNN = 145 ± 39 vs. 49 ± 20 vs. 46 ± 17ms; rMSSD = 39 ± 17 vs. 36 ± 20 vs. 21 ± 10 ms; pNN50 = 11 ± 9 vs. 15 ± 16 vs. 4 ± 6% from Holter vs. lie down vs. standing positions, respectively). HRV values of the ‘healthy’ group were higher than those for the ‘unhealthy’ group (SDNN = 49 ± 20 vs. 40 ± 18ms, lie down position; 46 ± 17 vs. 40 ± 18ms, standing position and 145 ± 139 vs. 132 ± 40ms from Holter measures, ‘healthy’ vs. ‘unhealthy’ respectively).

## Discussion

Our findings show reference values of HRV time domain measures on a rural sample using Holter monitor during daily activities. Variables that represent autonomic balance decreased in a linearly with increasing age. Interestingly, vagal tone associated variables had some particularities. The LOESS regression curves for rMSSD were U-shaped and pNN50 showed a reversal increase over 60y. Males had higher mean values than females, with statistical significance by age-decade groups. Race-related differences were not statistically significant. In a further analysis on a subset that underwent resting ECG, representatives of autonomic balance displayed a linear pattern of decrease with increasing age. Vagal tone representatives had an inverse pattern of increase with increasing age, specifically above 60y. rMSSD and pNN50 had a reversal increase over 60y in the standing protocol, but not in the lying protocol. Finally, time domain variables from the Holter had higher mean values than those from resting ECG. The ‘healthy’ group usually had higher values than the ‘unhealthy’ group. Because HRV measures are associated with worse cardiovascular outcomes, the knowledge about the reference values of the population may shed light on future disease prevention.

Sammito et al. [9] recently called attention to the fact that reference HRV values from the adult healthy population are still lacking and pointed out that the reference values differed from the values published in the 1996-Guidelines of the Task Force [1]. They studied almost 700 healthy subjects from 20 to 60y from a German population, using 24h-ECG measurement. They observed a decrease in HRV measures with increasing age, and sex-related differences [9]. Our findings are in line with those of the German population, i.e., HRV decreases with increasing age and sex-related differences exist. However, our population reference values are closer to the reference values stated in the 1996-Guidelines [1] than values stated by the Sammito study [9]. For instance, in the 1996-Guidelines [1], mean values are provided for SDNN (141 ± 39ms), SDANN (127 ± 35ms), and rMSSD (27 ± 12ms). Our population mean values were SDNN (146 ± 37ms), SDANN (130 ± 39ms), and rMSSD (41 ± 21ms). The Sammito et al. study [9] mentioned that the 50^th^ percentile for the SDNN was lower than the value from the 1996-Guidelines [1], and in contrast, the values for rMSSD and SDANN were higher than those published in the 1996-Guidelines [1]. Our rMSSD values are also higher than those published in the 1996-Guidelines [1], similarly to what Sammito and coworkers found in their study [9]. It seems that our reference values for SDNN and SDANN are closer to those stated in the 1996-Guidelines [1] and rMSSD and pNN50 are closer to those stated by the Sammito study [9]. The population phenotype may also play a role in HRV reference values.

Regarding the sex influence on HRV, several studies agree that sympathetic representatives are higher in males than females [7,9,17]. However, vagal representatives still need more clarification in relation to differences by sex, because they are shown to be higher in females than in males [7,17]. Our findings showed higher HRV values for males than females across almost all age-decade groups, but showed some unexpected patterns, especially for female’s age 40–49y and 50–59y showing higher values for females than males for the vagal representative measures. Due to the fact that HRV measures have been related to mental disease and emotional distress, such as depression [18,19,20] and we have not excluded those disorders in our apparently ‘healthy’ subset, some unknown factors may have contributed to the pattern of HRV measures, revealing sex differences only in specific, and not all, age-decade groups. Furthermore, population differences may influence the vagal patterns, because studies that show vagal measures higher in females than males had limited the age range of the sample (up to 60y [7] and up to 74y) [17], while our subset, even in small number, was predominantly female and up to 90y. In addition, animal studies suggest that estrogen influence is associated with higher vagal response in female than males [21]. Therefore, sex differences may be linked to age distribution and should be interpreted with caution by population differences.

Because aging is the main factor that impacts cardiac autonomic control, knowledge of the HRV patterns in elderly individuals may display unexpected findings. Almeida-Santos et al. [22] studied time domain variables of individuals (aged 40 to 100y), showing that global autonomic regulation (SDNN, SDANN, and SDNN-index) decreased linearly with age in both sexes. They found that the parasympathetic outflow markers (rMSSD and pNN50) had a U-shape in both sexes with the nadir in the seventh decade [22]. Likewise, our findings showed that rMSSD had a U-shaped pattern, but the nadir was around 53y. In addition, our findings on pNN50 showed a reverse increase over 60y, but not a U-shaped pattern. Because the authors found a similar U-shaped pattern in individuals with diabetes, but with lower values compared with subjects without [22], it seems that this U-shaped pattern, of the parasympathetic arm representatives, may be driven by both the aging process and disease influence.

Disease influence has already been studied in the context of hypertensive elderly subjects according to HRV measures [6]. Authors found that hypertensive elderly patients had decreased HRV values, especially decreased parasympathetic modulation compared with normotensive elderly [6]. However, the main limitations of this study [6] are the small sample size (n = 80), and that a Polar RS800CX heart rate monitor was used to record data for HRV analysis, not 24h-Holter-ECG measurements.

Because autonomic modulation in healthy elderly subjects showed an increase in sympathetic modulation and a decrease in vagal modulation after aerobic exercise [23,24], vagal tonus increases at a specific age may point out to autonomic dysfunction. Therefore, this reversal increase of vagal representatives above 60y, showed by our findings, in addition to the parasympathetic outflow U-Shape shown in the healthy elderly population (aged from 40y to 100y) study [22], may shed light on a pattern associated with autonomic dysfunction, not only the aging process. Indeed, aging modulates HRV, but patterns of change are measure dependent [23]. Zulfiqar et al. [10] described a parasympathetic aging pattern of reversal increase with change in the 8^th^ decade and reported this pattern as a sign of longevity. They stated that ‘persistently high HRV in the elderly represents a marker predictive of longevity’ [10]. Because low HRV is associated with cardiovascular diseases [2,3,4,24], it is plausible that the reversal increase of vagal tone HRV markers may be linked to longevity. However, higher levels of some traditional cardiovascular risk factors (non-HDL-cholesterol and C-reactive protein) were associated with lower total power, lower HF and LF measures [24]. Disease-related autonomic modulation is not restricted to the parasympathetic effect, but also to the sympathetic effect [24]. Therefore, in healthy subjects, the reversal increase should be shown not only for vagal tone HRV markers, but also for sympathetic ones if this increase is truly associated with longevity as stated before [10]. In summary, cardiac autonomic control in the aging process, with HRV patterns age-dependent and health-related, still needs clarification. Longitudinal studies may follow HRV patterns as markers of autonomic dysfunction driven by age-related or disease-dependent factors.

### Study limitations and strengths

Our study has limitations. First, the cross-sectional nature of this study restricts causal inference. Second, due to the small number of subjects in the group above 60 years, this limits conclusions for this age group, but it can generate hypotheses by the patterns shown here. Third, there was a lack of frequency domain HRV measures for the Holter, which would confirm some specific patterns especially found on vagal HRV associated variables. However, we did a further analysis with a resting ECG subset that had time and frequency domain HRV measures. In addition, to avoid noise in the data, we displayed LOESS regression curves with data from Holter, as well as data from resting ECG. Furthermore, we excluded the well-known factors that affect HRV measures, and restricted our analysis to those reported as ‘healthy.’ Finally, participants who underwent Holter and resting ECG did the exams in the same time period (all in the second-wave evaluation period); therefore, time-related measurement differences could be avoided.

## Conclusions

In this rural sample, using Holter, our results are in line with findings from non-rural areas, pointing out that HRV measures in the time domain differ by sex and age-decade groups, showing higher mean values for males than for females. Our findings also reveal unexpected slope patterns for vagal tone representatives, such as the U-shaped pattern for rMSSD and reversal increase above 60y for pNN50. Autonomic balance representatives (SDNN and SDANN) decreased linearly with increasing age. In addition, this study also highlights that the resting ECG may not be sensitive enough for gathering some vagal tone patterns. In summary, our study showed a U-shaped and reversal increase patterns in parasympathetic variables, even in a young and healthy sample, which may point to early autonomic dysfunction. Longitudinal studies are needed to certify the association of HRV patterns and disease related.

## Data Accessibility Statements

Researchers can apply for data and biomaterial by submitting a proposal to the principal investigator, ACP (alexandre.pereira@incor.usp.br).

## Additional File

The additional file for this article can be found as follows:

10.5334/gh.873.s1 Suplemental Material. Appendix A.

## Abbreviations

**HRV** = heart rate variability

**HF nu** = high-frequency power spectrum from 0.15 to 0.40 Hz/normalized values

**LF nu** = low-frequency power spectrum from 0.04 to 0.15 Hz/normalized values

**LF/HF** = low-frequency high-frequency ratio

**LOESS** = Locally Weighted Scatterplot Smoothing

**pNN50** = percentage of consecutive NN intervals that deviate from one another by more than 50 ms.

**rMSSD** = root mean square of successive difference of NN intervals

**SDANN** = standard deviation of the average of all consecutive 5-minute NN intervals

**SDNN** = standard deviation of all normal-to-normal intervals
