# Supplementary material for: Age and Sex Differences in Heart Rate Variability and Vagal Specific Patterns – Baependi Heart Study
Source: Glob Heart. 2020 Oct 21;15(1):71. doi: 10.5334/gh.873 (PMC7583712; doi:10.5334/gh.873)
Supplement: Suplemental Material. — Appendix A. [file gh-15-1-873-s1.pdf]

Suplemental Material - Appendix A

Table A1. Characteristics of the Full Sample (all complete 24hour-Holter ECG) at the second wave evaluation period (from 2010 to 2016)

	**Full sample (24hour-Holter ECG) n= 1,287**
**Age, y**	48 ±16
**Sex (Male), %**	37
**Race, %**	
White	71
Black	7
Other	22
**Smoking, %**	
Never	67
Former	20
Current	13
**BMI, kg/m^2**	26 ±5
**Waist circumference, cm**	92 ±13
**Hip circumference, cm**	100 ±11
**Neck circumference, cm**	36 ±4
**Total cholesterol, mg/dL**	20 ±42
**LDL-chol, mg/dL**	124 ±36
**HDL-chol, mg/dL**	47 ±11
**Triglycerides, mg/dL**	143 ±83
**Glucose, mg/dL**	94 ±23
**HbA1c, %**	5.60 ±0,90
**Creatinine, mg/dL**	0.87 ±0.20
**HTN, %**	36
**DM, %**	7
**Mean 24h-Systolic BP, mmHg**	118 ±11
**Mean 24h-Diastolic BP, mmHg**	73 ±8
**Medication, %**	
Antihypertensive	31
Oral hypoglicemic	6
Statin	12
BB use	9

Data are shown as mean ±SD for continuous and percentage for categorical variables. ECG= electrocardiogram, BMI= body mass index, chol= cholesterol; LDL= low-density lipoprotein; HDL= high-density lipoprotein; HbA1c= glycated hemoglobin; HTN= hypertension; DM= diabetes Mellitus; BP= blood pressure.; BB= beta blocker.

Table A2. 24 Hour-Holter ECG Characteristics

	"Healthy" sample (n= 543)	Full sample (24-hour-Holter ECG) (n= 1,287)
**Min HR, bpm**	50±7	51 ±7
**Mean HR, bpm**	79±9	78 ±9
**Max HR, bpm**	144±20	139 ±231
**NN intervals, nº**	97,166 ±15,934	95,462 ±17,382
**mean NN intervals, ms**	786±93	796 ±100
**SDNN, ms**	146±37	138 ±41
**SDANN, ms**	130±39	123 ±39
**SDNN index, ms**	64±20	59 ±25
**rMSSD, ms**	41±21	40 ±27
**pNN50, %**	12±10	10 ±10
**TINN, ms**	612±174	567 ±181

Data are shown as mean±SD. "Healthy" sample=excluded those under medication use, under BB use, obese (BMI ≥30kg/m^2), current smokers and diseases, such as HTN, DM, stroke, acute myocardial infarction, cancer, cardiac revascularization or angioplasty (see Appendix Figure A1). HR=heart rate; NN intervals = normal-to-normal intervals; SDNN=standard deviation of NN intervals; SDANN=standard deviation of the average of all consecutive 5-minute NN intervals; SDNN index=mean of the standard deviations of all normal sinus NN intervals for all 5-min segments; rMSSD=root mean square of successive difference of NN intervals; pNN50=percentage of consecutive NN intervals that deviate from one another by more than 50 ms; TINN=triangular interpolation of NN intervals; AFib=atrial fibrillation. No AFib patients and pacemaker subjects were in this subset.

Table A3. Univariate Restricted Analysis of all Complete Data (resting ECG 10' protocol and 24h-Holter ECG) and Time Domain Measures by Groups

**Total (N=1194)**	**Healthy (n=513)**	**Unhealthy (n=681)**	***P*-value**
**Lie down 10'**			
**SDNN, ms**	49±20	40±18	**<0.001**
**rMSSD, ms**	36±20	30±19	**<0.001**
**pNN50, %**	15±16	11±15	**<0.001**
**Standing 10'**			
**SDNN, ms**	46±17	40±18	**<0.001**
**rMSSD, ms**	21±10	21±14	0.638
**pNN50, %**	4±6	4±8	0.180
**Time domain HRV measures from 24h-hour ECG Holter**			
**SDNN, ms**	145±39	132±40	**<0.001**
**rMSSD, ms**	39±17	39±28	0.940
**pNN50, %**	11±9	9±10	**<0.001**

Data are shown as mean ±SD for continuous variables. *P*-value by independent *t* test for continuous variables. ECG= electrocardiogram; HRV= heart rate variability; NN= normal-to-normal; SDNN= standard deviation of NN intervals; rMSSD= root mean square of successive difference of NN intervals; pNN50= percentage of consecutive NN intervals that deviate from one another by more than 50 ms;

Figure A1. Exclusion criteria for the sample

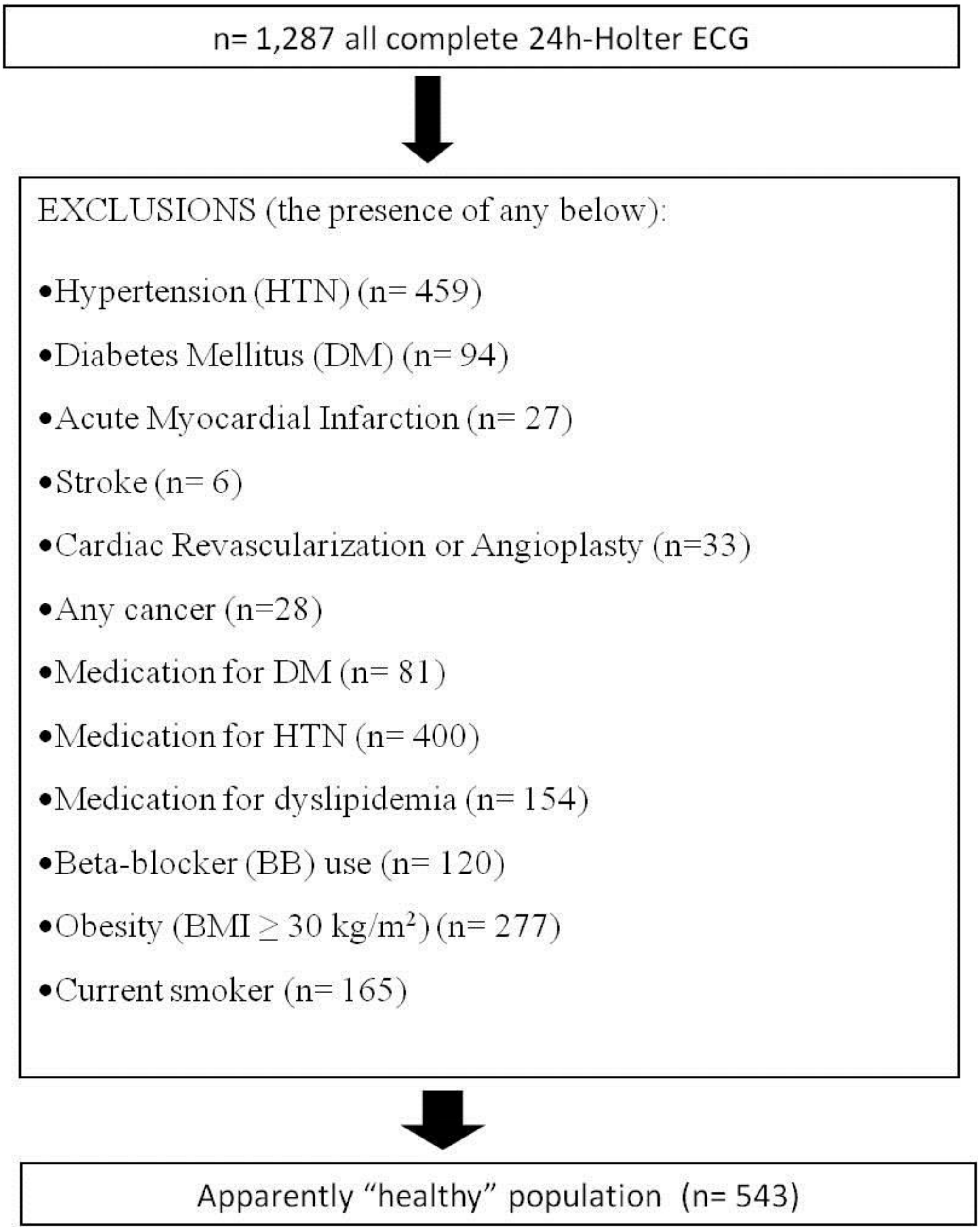

Figure A2. HRV measures showing mean values by age-decade and race distribution of the “Healthy” sample (n=543)

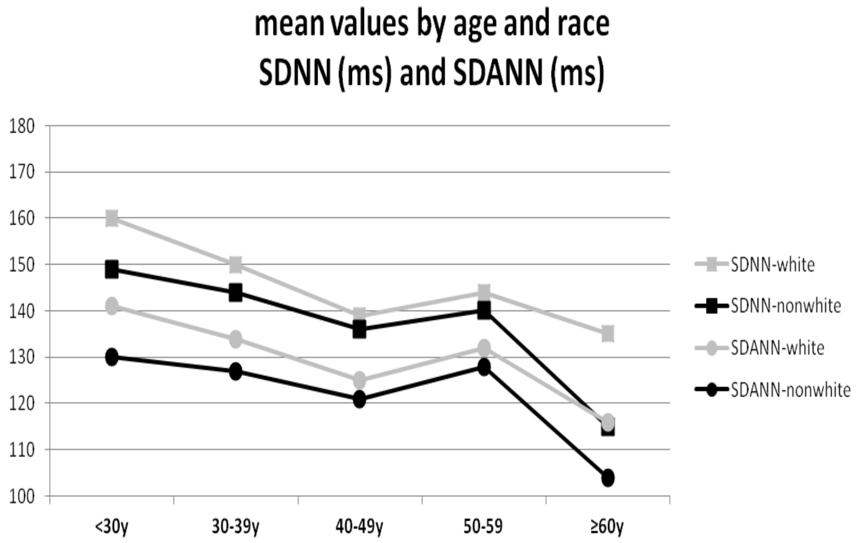

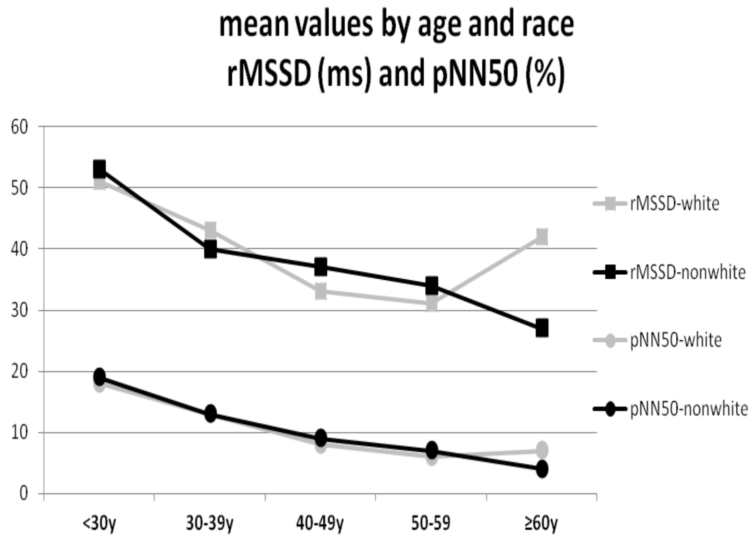

SDNN= standard deviation of NN intervals; SDANN= standard deviation of the average of all consecutive 5-minute NN intervals; rMSSD= root mean square of successive difference of NN intervals; pNN50= percentage of consecutive NN intervals that deviate from one another by more than 50 ms. Race (white versus non-white) white (n= 402) and non-white (n= 141). Age ≥18 and < 30y (n= 137); age 30-39y (n= 154); age 40-49y (n= 110); age 50-59y (n= 92); age ≥60y (n= 50). According to 2-way ANOVA, testing difference among groups, for SDNN (p-value for age decade=0.003, for race= 0.070 and for race*agedecade interaction=0.870); for SDANN (p-value for age decade= 0.030; p-value for race= 0.100, and for race*agedecade interaction= 0.955); for rMSSD (p-value for age decade= <0.001, for race=0.310, and for agedecade*race interaction= 0.383); for pNN50 (p-value for age decade= < 0.001, for race= 0.916, and for agedecade*race interaction= 0.962).

Figure A3. HRV measures (SDNN and SDANN) stratified by sex and mean values by age-decade and race distribution of the "Healthy" sample (n=543)

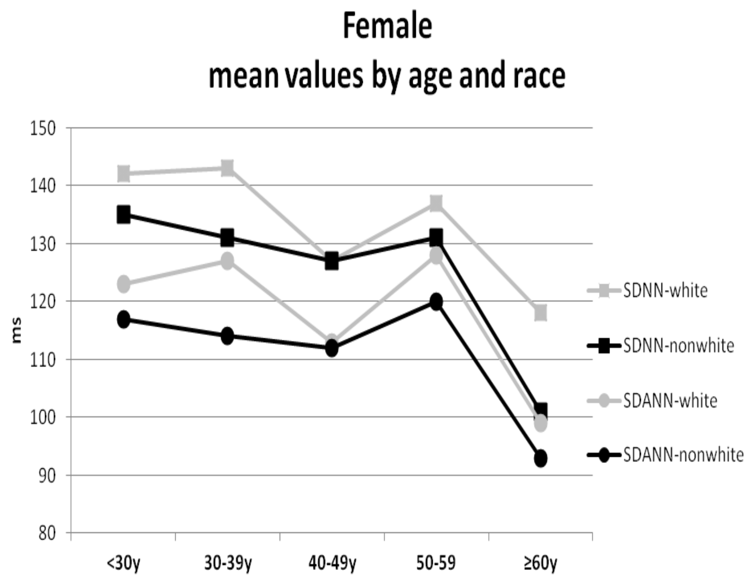

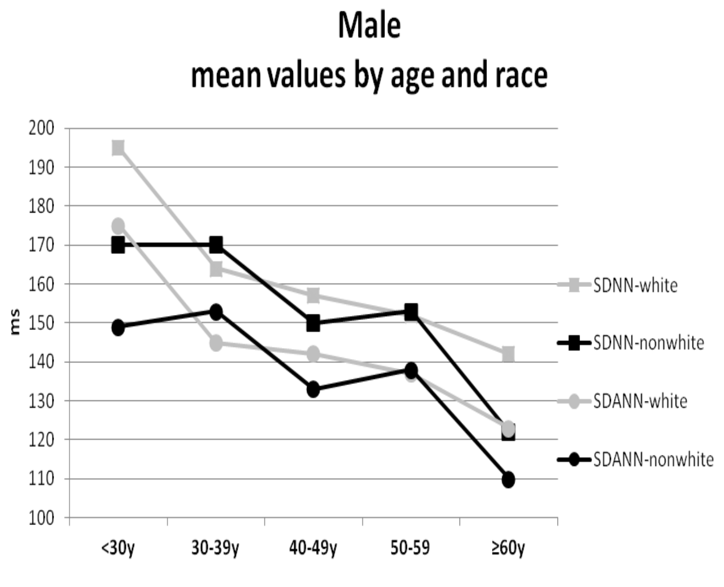

NN intervals= normal-to-normal intervals; SDNN= standard deviation of NN intervals; SDANN= standard deviation of the average of all consecutive 5-minute NN intervals; Female (n =318) and Male (n= 225); Race (white versus non-white). Female white (n= 233) and female nonwhite (n= 85). Male white (n= 169) and male nonwhite (n=56). Age ≥18 and < 30y (n= 137); age 30-39y (n= 154); age 40-49y (n= 110); age 50-59y (n= 92); age ≥60y (n= 50). Stratified analysis for SDNN (p-value for interaction agedecade*race for female = 0.823 and for p-value for male = 0.538). P-value testing interaction (agedecade* race* sex) for SDNN = 0.434. Stratified analysis for SDANN (p-value for interaction agedecade*race for female = 0.832 and for male = 0.482). P-value testing interaction (agedecade*race*sex) for SDANN= 0.307.

Figure A4. HRV measures (rMSSD and pNN50) stratified by sex and mean values by age-decade and race distribution of the "Healthy" sample (n=543)

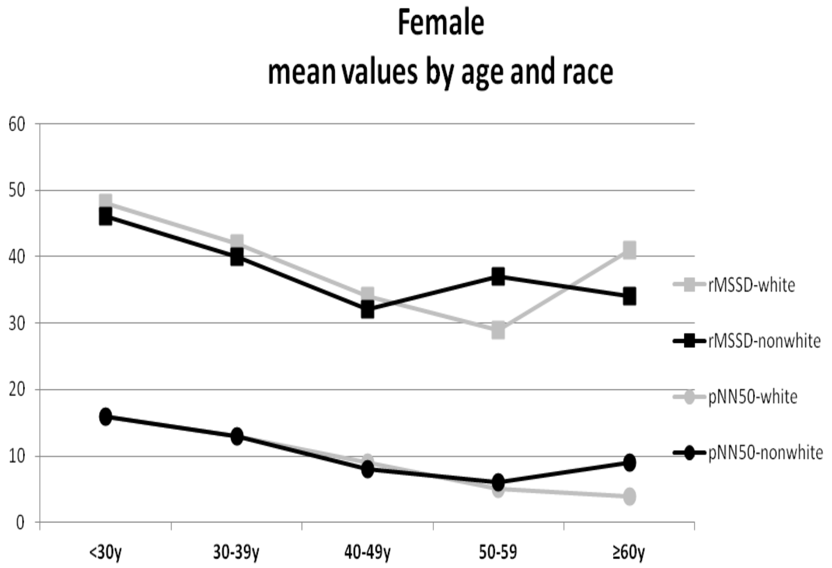

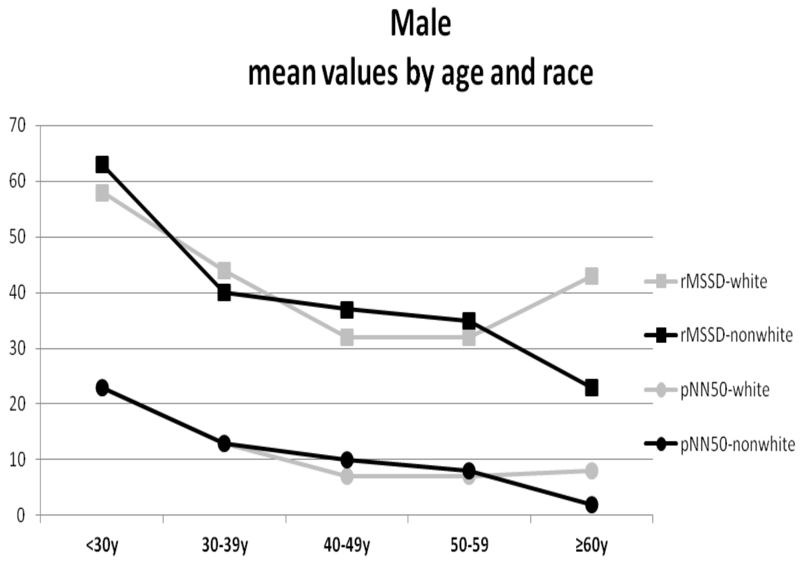

NN intervals= normal-to-normal intervals; rMSSD= root mean square of successive difference of NN intervals (unit= ms); pNN50= percentage of consecutive NN intervals that deviate from one another by more than 50 ms (unit=%). Female (n =318) and Male (n= 225); Race (white versus non-white). Female white (n= 233) and female nonwhite (n= 85). Male white (n= 169) and male nonwhite (n=56). Age ≥18 and < 30y (n= 137); age 30-39y (n= 154); age 40-49y (n= 110); age 50-59y (n= 92); age ≥60y (n= 50). Stratified analysis for rMSSD (p-value for interaction agedecade*race for female =0.894 and p-value for male= 0.421). P-value testing interaction (agedecade*race*sex) for rMSSD= 0.763. Stratified analysis for pNN50 (p-value for interaction agedecade*race for female = 0.921 and p-value for male = 0.748). P-value testing interaction (agedecade*race*sex) for pNN50 = 0.657.

Figure A5. Time domain HRV measures of the rest ECG 10 min protocol sample showing mean values for lie down and standing positions.

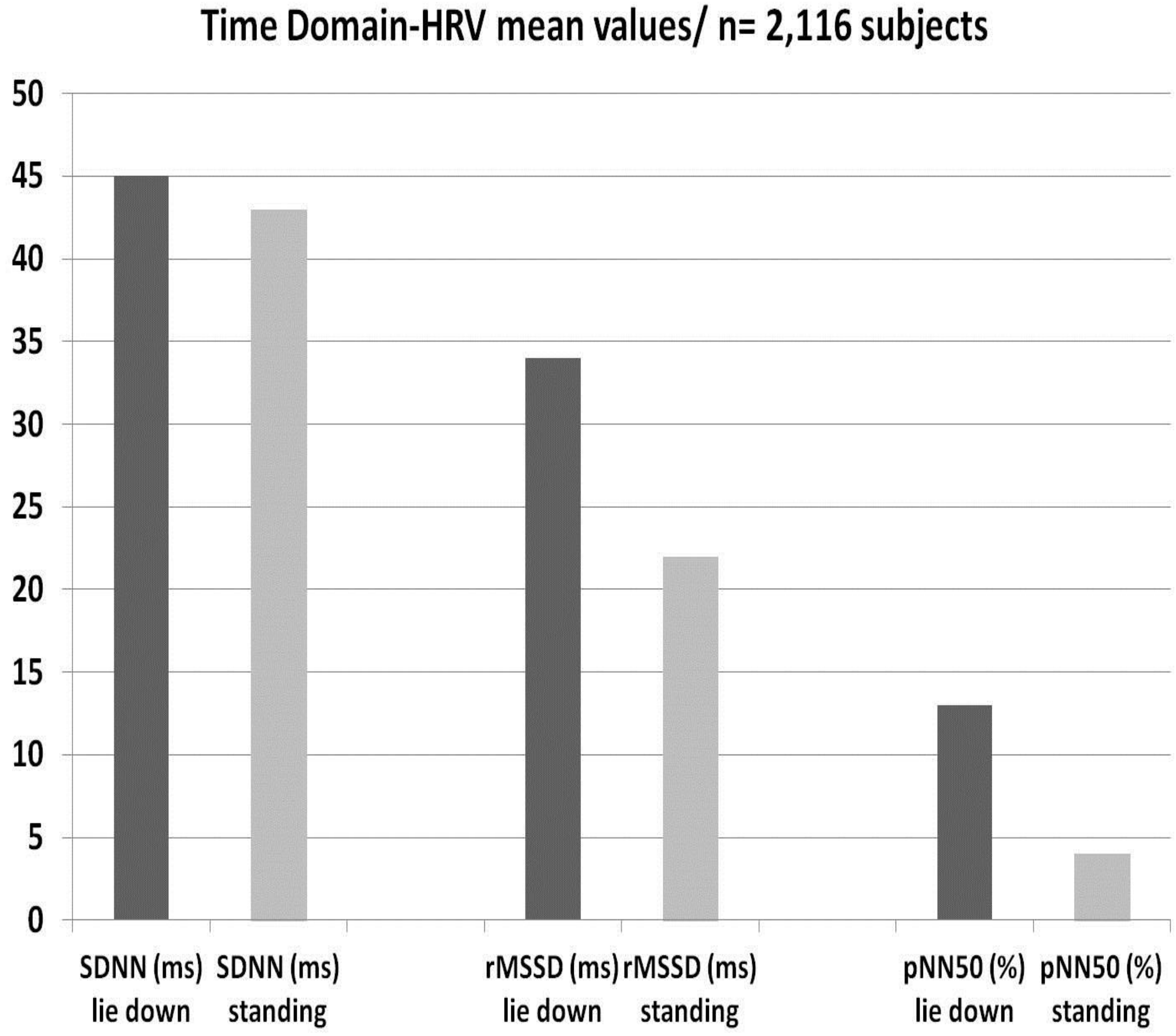

HRV= heart rate variability; SDNN index = mean of the standard deviations of all normal sinus NN intervals for all 5-min segments; rMSSD= root mean square of successive difference of NN intervals; pNN50= percentage of consecutive NN intervals that deviate from one another by more than 50 ms;

Figure A6. Frequency domain HRV measures of the rest ECG 10 min protocol sample showing mean values for lie down and standing positions.

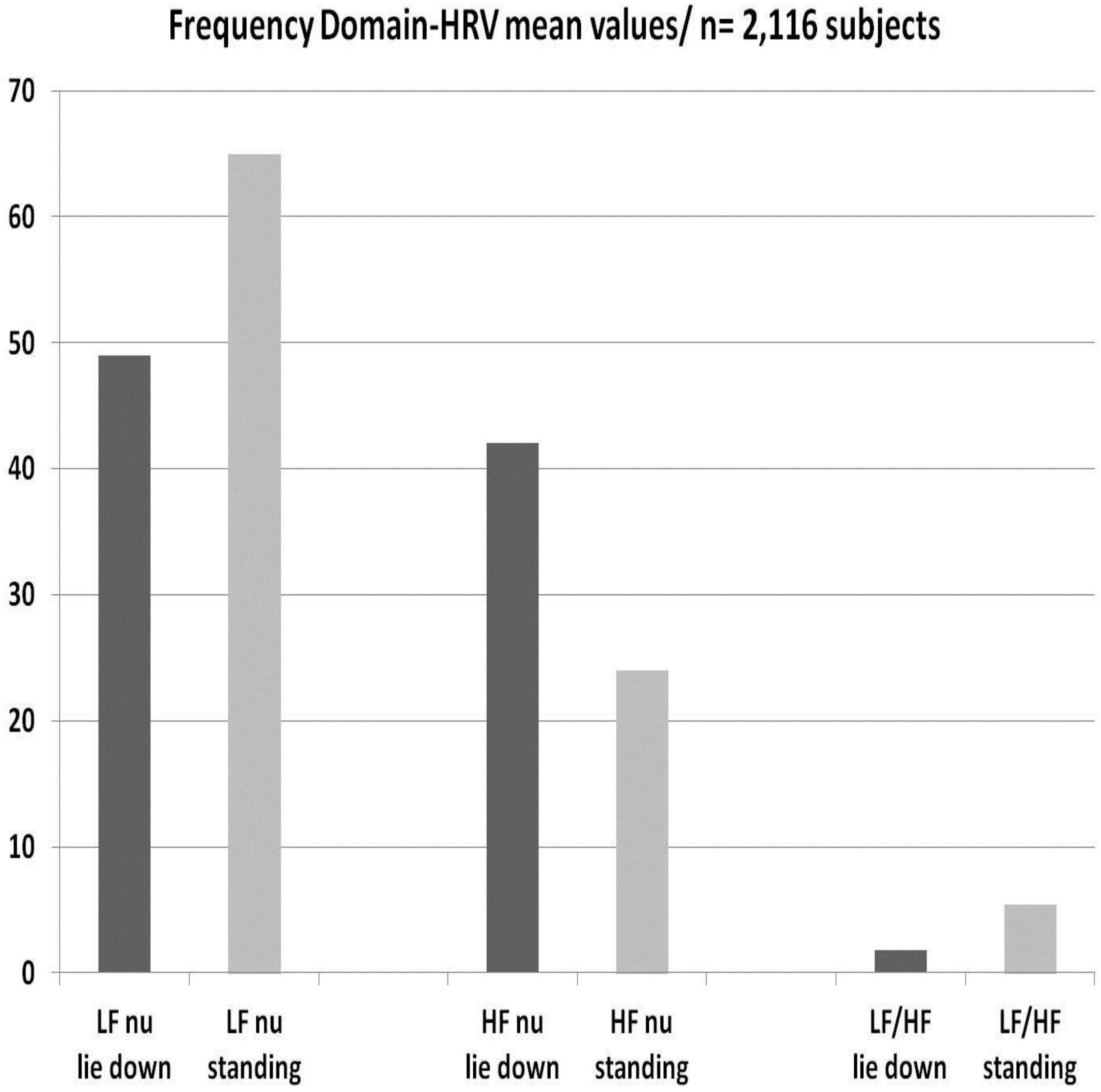

HRV= heart rate variability; LF= low-frequency power spectrum from 0.04 to 0.15 Hz; HF= high-frequency power spectrum from 0.15 to 0.40 Hz; LF/HF= low-frequency high-frequency ratio; nu= normalized units.

Figure A7. Graphic display of the mean values by age-decade groups of the sample with rest ECG 10 min protocol (n=2,116), while lie down and standing positions.

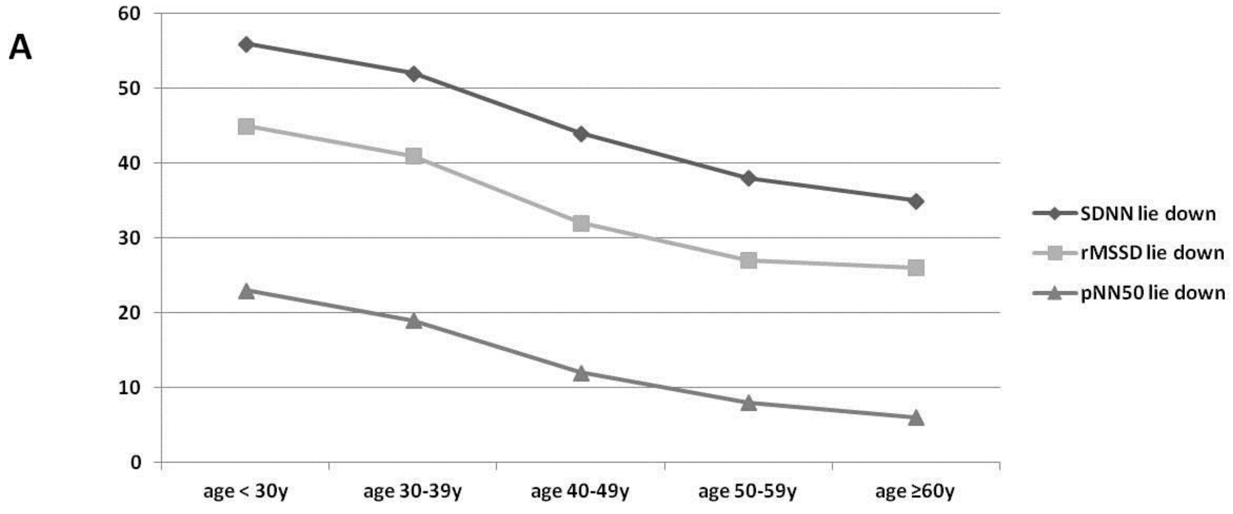

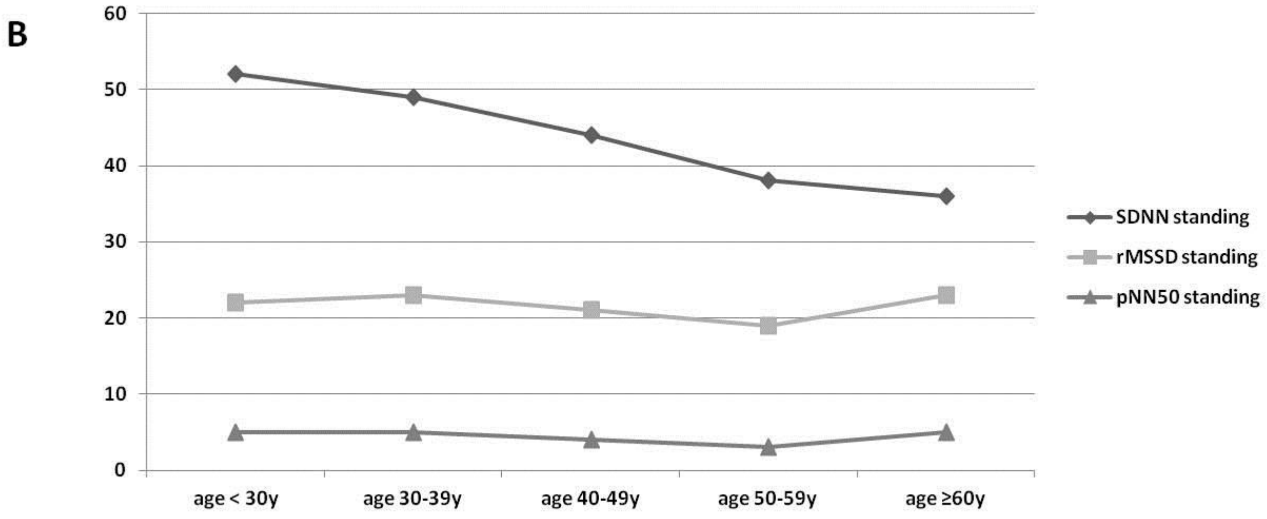

SDNN= standard deviation of NN intervals (unit= ms); rMSSD= root mean square of successive difference of NN intervals (unit= ms); pNN50= percentage of consecutive NN intervals that deviate from one another by more than 50 ms (unit= %). A represents rest ECG 10 min lie down position and B represents rest ECG 10 min standing position.

Figure A8. Graphic display of the mean values by age-decade groups of the sample with rest ECG 10 min protocol (n=2,116), while liedown and standing positions.

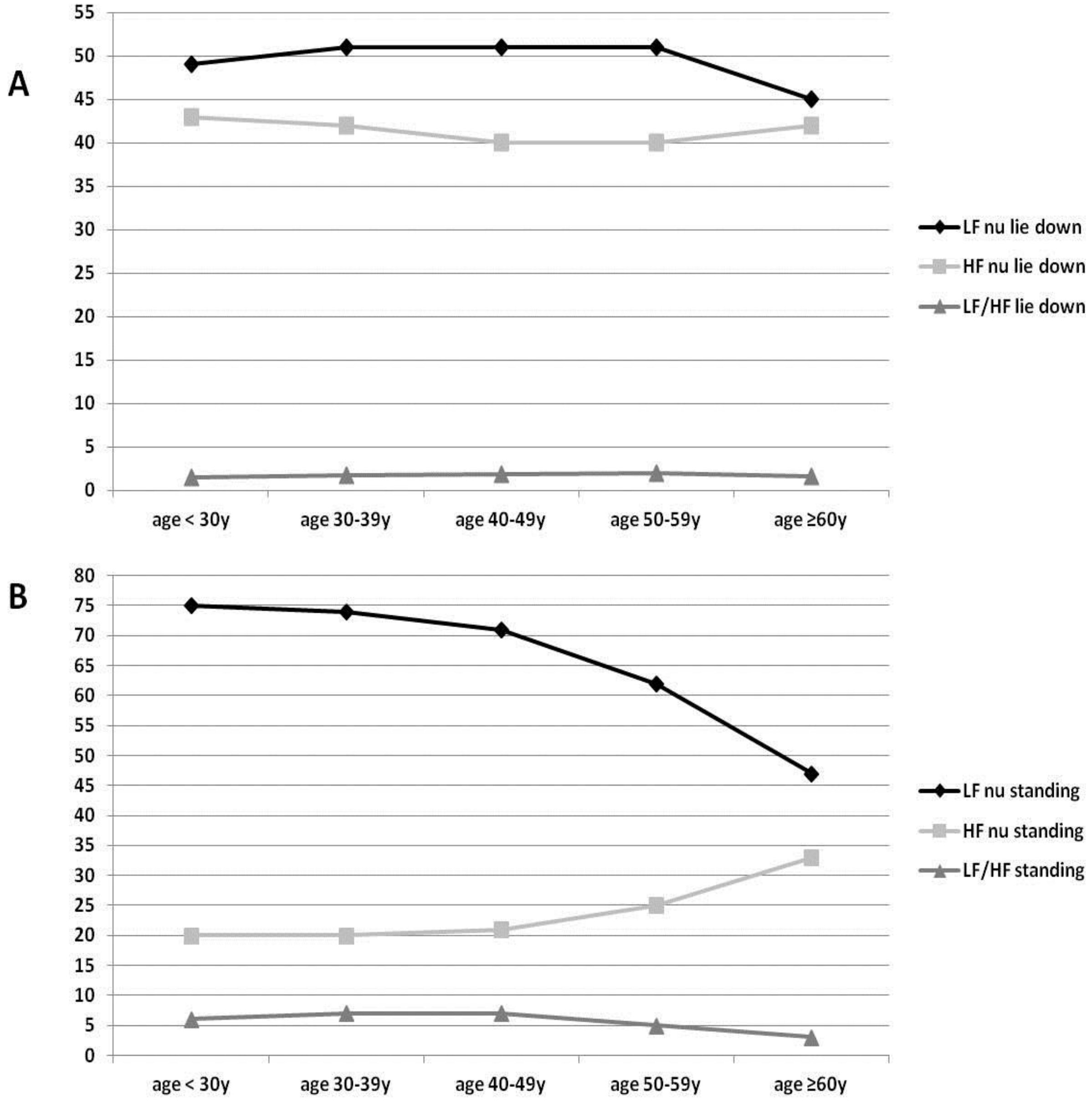

HRV= heart rate variability; LF= low-frequency power spectrum from 0.04 to 0.15 Hz; HF= high-frequency power spectrum from 0.15 to 0.40 Hz; LF/HF= low-frequency high-frequency ratio; nu= normalized units. A represents rest ECG 10 min lie down position and B represents rest ECG 10 min standing position.

Figure A9. LOESS regression curves (SDNN) of “healthy” population from samples with rest ECG 10 min protocol and for 24h-Holter ECG

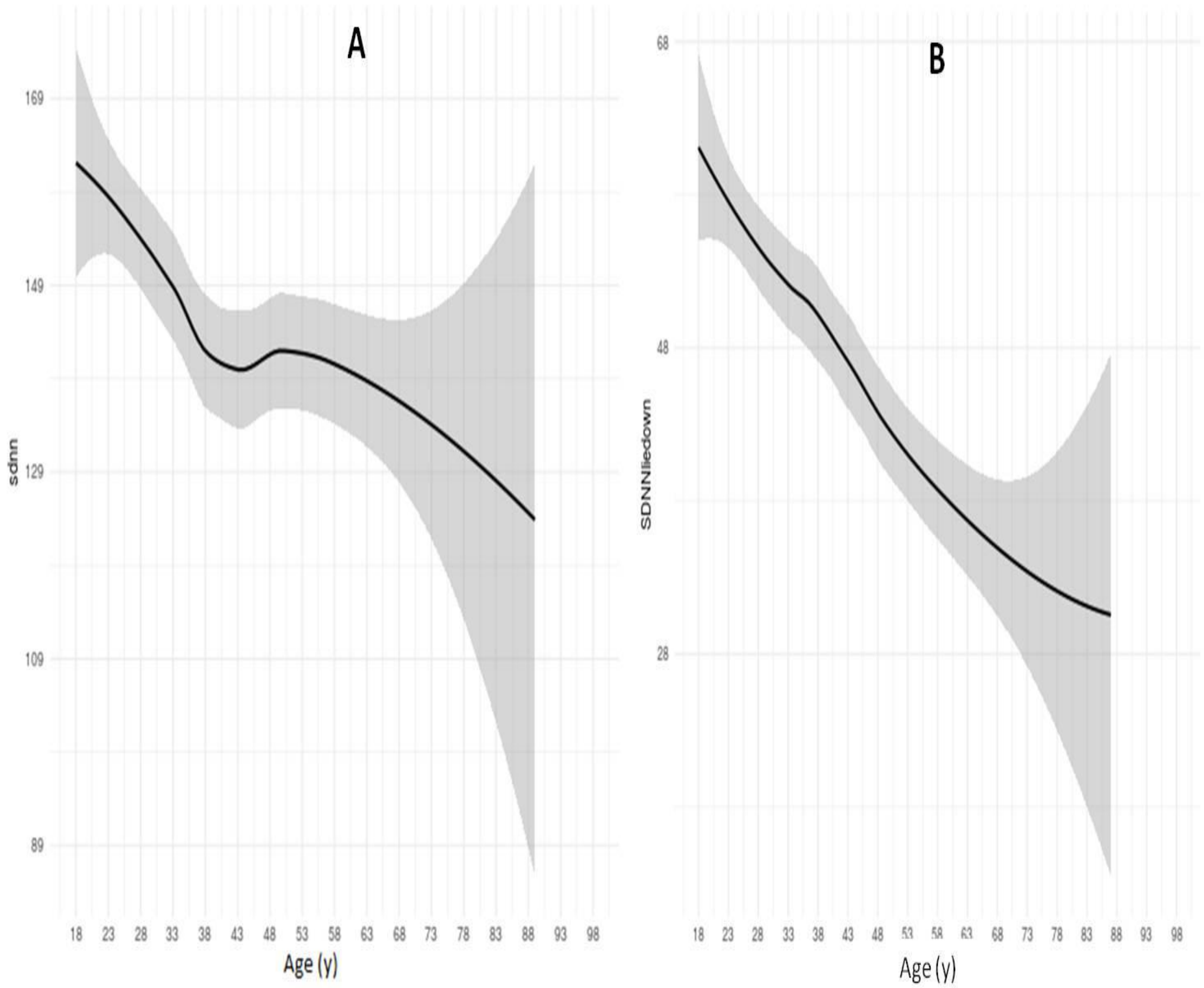

SDNN= standard deviation of NN intervals. SDNN is measured in milliseconds. A represents the healthy sample underwent to 24h-Holter ECG (n=543) and B represents the healthy sample underwent to rest ECG 10 min protocol (n= 538).

Figure A10. LOESS regression curves (rMSSD) of "healthy" population from samples with rest ECG 10 min protocol and for 24h-Holter ECG

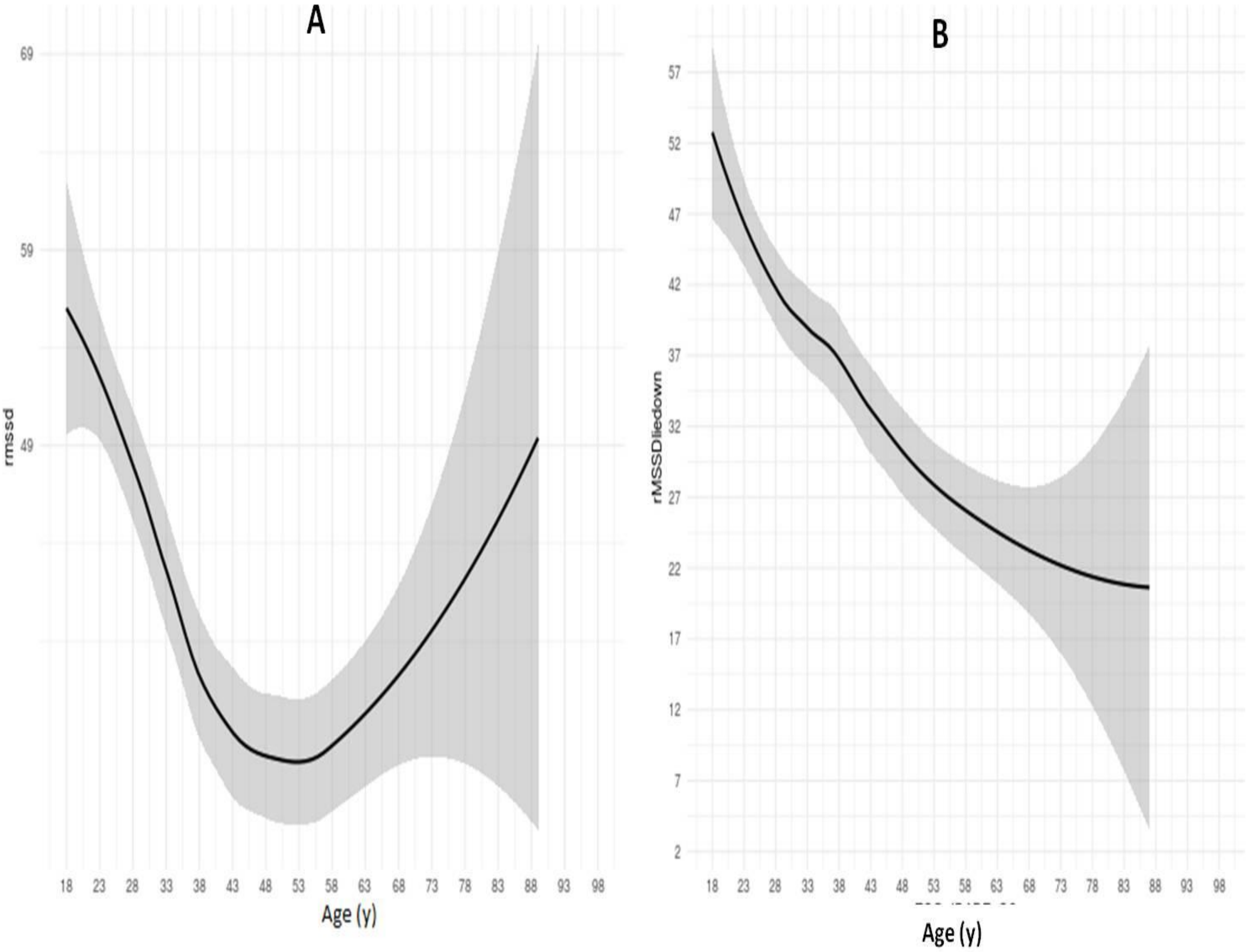

rMSSD= root mean square of successive difference of NN intervals; rMSSD is measured in milliseconds. A represents the healthy sample underwent to 24h-Holter ECG (n=543) and B represents the healthy sample underwent to rest ECG 10 min protocol (n= 538).

Figure A11. LOESS regression curves (pNN50) of "healthy" population from samples with rest ECG 10 min protocol and for 24h-Holter ECG

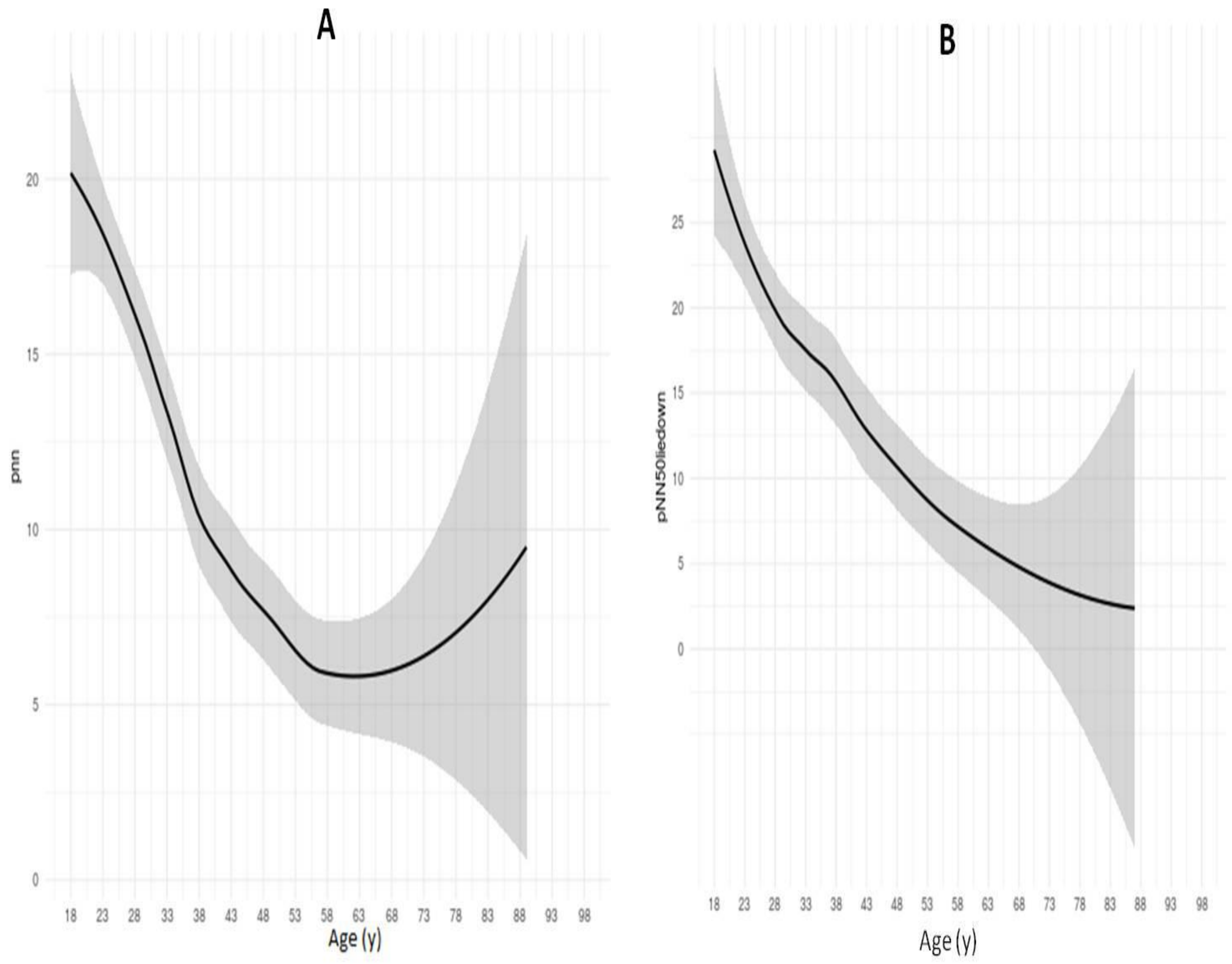

pNN50= percentage of consecutive NN intervals that deviate from one another by more than 50 ms. pNN50 is measured in percentage. A represents the healthy sample underwent to 24h-Holter ECG (n=543) and B represents the healthy sample underwent to rest ECG 10 min protocol (n= 538).
